# Bowel movement frequency and risk of colorectal cancer in a large cohort study of Japanese men and women

**DOI:** 10.1038/sj.bjc.6601735

**Published:** 2004-03-09

**Authors:** M Kojima, K Wakai, S Tokudome, K Tamakoshi, H Toyoshima, Y Watanabe, N Hayakawa, K Suzuki, S Hashimoto, Y Ito, A Tamakoshi

**Affiliations:** 1 Department of Health Promotion and Preventive Medicine, Nagoya City University Graduate School of Medical Sciences, 1 Kawasumi, Mizuho-cho, Mizuho-ku, Nagoya 467-8601, Japan; 2 Division of Epidemiology and Prevention, Aichi Cancer Center Research Institute, 1-1 Kanokoden, Chikusa-ku, Nagoya 464-8681, Japan; 3 Department of Preventive Medicine/Biostatistics and Medical Decision Making, Nagoya University Graduate School of Medicine, 65 Tsurumai-cho, Showa-ku, Nagoya 466-8550, Japan; 4 Department of Public Health/Health Information Dynamics, Nagoya University Graduate School of Medicine, 65 Tsurumai-cho, Showa-ku, Nagoya 466-8550, Japan; 5 Department of Epidemiology for Community Health and Medicine, Kyoto Prefectural University of Medicine Graduate School of Medical Science, Kawaramachi-Hirokoji, Kamigyo-ku, Kyoto 602-8566, Japan; 6 Department of Epidemiology, Research Institute for Radiation Biology and Medicine, Hiroshima University, 1-2-3, Kasumi, Minami-ku, Hiroshima 734-8553, Japan; 7 Department of Public Health, Fujita Health University School of Health Sciences, 1-98 Dengakugakubo, Kutsukake-cho, Toyoake, Aichi 470-1192, Japan; 8 Department of Hygiene, Fujita Health University School of Medicine, 1-98 Dengakugakubo, Kutsukake-cho, Toyoake, Aichi 470-1192, Japan

**Keywords:** colorectal carcinoma, constipation, diarrhoea, laxative, prospective study

## Abstract

The relationship between bowel movement (BM) frequency and the risk of colorectal cancer was examined in a large cohort of 25 731 men and 37 198 women living in 24 communities in Japan. At enrolment, each participant completed a self-administrated questionnaire on BM frequency and laxative use. Incidence rate ratios (IRR) with 95% confidence intervals (CI) were estimated using Cox's proportional-hazard model. During the follow-up period (average length 7.6 years), 649 cases of colorectal cancer, including 429 cases of colon cancer, were identified. Among women, subjects who reported a BM every 2–3 days had the lowest risk of developing colorectal (IRR=0.71, 95% CI=0.52–0.97) and colon cancer (IRR=0.70, 95% CI=0.49–1.00), whereas those reporting a BM every 6 days or less had an increased risk of developing colorectal (IRR=2.47, 95% CI=1.01–6.01) and colon cancer (IRR=2.52, 95% CI=0.93–6.82) compared with those reporting ⩾1 BM per day. A similar, but nonsignificant, association between the frequency of BM and cancer risk was observed in men. There was no association between colorectal or colon cancer risk and laxative use. Regulating BM frequency might therefore have a role in the prevention of colorectal cancer.

An association between constipation and the risk of colorectal cancer has long been noted. Prolonged intestinal transit time might not only increase the duration of contact between carcinogens in the stools and the gut wall, but could also concentrate carcinogens by increasing colonic water absorption (Sonnenberg and Müller, 1993). A meta-analysis of 14 case–control studies that examined the association between constipation or infrequent bowel movements (BMs) and colorectal cancer and found a statistically significant 48% increase in the pooled odds ratios for colorectal cancer in association with constipation. Recent case–control studies have also reported a relatively consistent positive relationship between constipation and colorectal cancer (Kotake *et al*, 1995; Le Marchand *et al*, 1997; Ghadirian *et al*, 1998; Jacobs and White, 1998; Roberts *et al*, 2003).

Since bowel habits might be influenced by the presence of colorectal cancer, retrospective studies cannot exclude the effects of the cancer itself, as well as recall bias, on their results. However, few prospective studies have addressed this issue. The only cohort study, which had a 12-year follow-up period involving 84 577 women, of colorectal cancer incidence and BM frequency or laxative use reported negative results (Dukas *et al*, 2000). The influence of BMs on male colorectal cancer has not been previously studied prospectively.

We conducted a large cohort study to investigate the association between bowel habits ‘laxative use’ susceptibility to diarrhoea and the colorectal cancer risk in Japanese men and women.

## MATERIALS AND METHODS

All data were taken from the Japan Collaborative Cohort (JACC) Study, the methods of which have been described in detail elsewhere (Ohno and Tamakoshi, 2001). Briefly, the original study population consisted of 110 792 Japanese adults aged 40–79 years. Enrolment began in 1988 and continued until the end of 1990 in 45 areas across Japan. Most subjects were recruited from the general population or when undergoing routine health checks in the municipalities. Written informed consent for participation was obtained individually from subjects, with the exception of a few study areas in which informed consent was provided at the group level after explaining the aims of the study and confidentiality of the data to community leaders. The study protocol was approved by the Ethics Committee of Medical Care and Research of the Fujita Health University School of Medicine, Japan.

Analyses were restricted to data from the 65 184 participants who lived in the 24 study areas in which cancer registries were available. A further 58 subjects with a previous history of colorectal cancer, and 2197 subjects for whom information about bowel habits was not available, were excluded. Therefore, a total of 62 929 individuals (25 731 men and 37 198 women) were involved in this analysis.

All participants completed a self-administered questionnaire on enrolment. This covered demographic characteristics and lifestyle factors such as diet, tobacco smoking, alcohol consumption, physical activity, BM frequency, susceptibility to diarrhoea and laxative use over the past year. The alternative answers provided on the questionnaire for the frequency of BM were: ‘daily’, ‘every 2–3 days’, ‘every 4–5 days’ and ‘every 6 days or less’. With regard to laxative use, the questionnaire asked only whether the participants used laxatives in the past one year at the time of enrolment; additional data on the type of laxative, the reason for use and the duration of use were not collected. Participants also provided information about susceptibility to diarrhoea by answering ‘yes’, ‘no’ or ‘neutral’ in response to the question: do you often have diarrhoea?

Population registries in the municipalities were used to determine the vital and residential status of subjects. Registration of death is required under the Family Registration Law in Japan, which applies throughout the country. Incidences of cancer were confirmed using records from the population-based cancer registries, which were supplemented by a systematic review of death certificates (Ohno and Tamakoshi, 2001); in some areas, medical records were also reviewed in major local hospitals. The mortality-to-incidence ratio for colorectal cancer was 0.28 in the cohort covered by the cancer registries. This figure is comparable with those calculated in the most accurate population-based cancer registries in Japan (Parkin *et al*, 2003), which indicates that most cases of colorectal cancer were identified in the study population.

The follow-up period ran from the time of the baseline survey through to the end of 1997 in all but three areas (in which it ran until the end of 1994, 1995 and 1996, respectively). The end point of the study was defined as the incidence of colorectal cancer (10th Revision of the International Classification of Diseases, ICD-10: C18–C20) or colon cancer (ICD-10: C18). The risk of rectal cancer was not analysed separately because of the relatively small number of cases observed. Subjects who moved out of the study area or died from causes other than colorectal cancer were treated as censored cases. During the study period, only 3.3% (2071) of the participants were lost from the follow-up as a result of a change of residence.

All analyses were carried out by sex using the SAS statistical package release 8.2 (SAS Inc., Cary, NC, USA). Differences in baseline characteristics between categories of BM frequency were tested using the chi-squared (*χ*^2^) test or one-way analysis of variance (ANOVA). The follow-up period for each participant was calculated as the time between completing the questionnaire and either the diagnosis of colon or rectal cancer, death, moving out of the study area or the end of the study – whichever occurred first.

The incidence rate ratios (IRR) and 95% confidence intervals (CI) for colorectal and colon cancer were estimated, by sex, using Cox's proportional-hazard model according to the levels of BM frequency, laxative use and susceptibility to diarrhoea. The categories of ‘every day’ for BM, ‘nonuse’ for laxative use and ‘no’ or ‘neutral’ for susceptibility to diarrhoea were used as reference groups.

Analyses were adjusted for the following potential confounding factors: age (continuous variable); body mass index (BMI) calculated as weight (kg) [height (m)]^−2^ and categorised as ‘⩾25 kg m^−2^’ or ‘<25 kg m^−2^’; intake frequency of green leafy vegetables (‘daily’ or ‘not daily’); intake frequency of alcohol (‘⩾5 days per week’ or ‘<5 days per week’); current smoking status (‘smoker’ or ‘nonsmoker’); time spent walking per day (‘⩽30 min or ‘>30 min’); history of colorectal cancer in parents or siblings (‘yes’ or ‘no’); and age at leaving full-time education (‘⩾20 years’ or ‘<20 years’). For each covariate, missing values were treated as an additional category and were included in the model. To determine the influence of symptoms of colorectal cancer on bowel habits, analyses were repeated excluding the first 3 years of follow-up. In all cases, two-sided *P*-values <0.05 were considered to be statistically significant.

## RESULTS

Within the study group, 1.1% of men and 4.0% of women reported infrequent BMs (every 4 days or less). The use of laxatives was more common among women (14.7%) than men (6.9%), whereas men were more likely to report frequent diarrhoea (20.3%) than were women (9.7%).

Table 1

**Table 1:** Background characteristics of the participants at baseline by BM frequency by sex

	**BM frequency**									
	**Men**					**Women**				
**Variable**	**⩾1per day, (*n*=22 930)**	**Every 2–3 days, (*n*=2526)**	**Every 4–5 days, (*n*=222)**	**Every 6 days or less, (*n*=53)**	***P*-value^a^**	**⩾1per day, (*n*=25 884)**	**Every 2–3 days, (*n*=9813)**	**Every 4–5 days, (*n*=1238)**	**Every 6 days or less, (*n*=263)**	***P* value^a^**
*Age (years)*										
Mean	57.6	59.7	62.5	65.9	<0.0001	58.4	57.3	57.4	58.8	<0.0001
s.d.	10.2	11.2	11.3	11.6		9.9	10.5	10.8	11.0	
										
*BMI (kg m*^−*2*^)										
Mean	22.7	22.2	21.7	21.7	<0.0001	23.1	22.6	22.5	22.0	<0.0001
s.d.	3.0	2.9	3.1	3.6		3.6	3.0	3.1	3.0	
										
Having green leafy vegetables every day (%)	26.2	26.1	20.7	22.6	0.29	32.5	27.8	27.1	24.7	<0.0001
										
Daily alcohol drinking (%)	48.4	36.0	33.3	28.3	<0.0001	5.2	4.7	4.3	4.9	0.15
										
Current smokers (%)	50.3	48.9	51.8	47.2	0.55	4.5	4.7	8.1	11.4	<0.0001
										
Daily walking time <30 min (%)	25.9	32.3	38.3	50.9	<0.0001	22.4	26.6	31.9	37.3	<0.0001
										
Having family history of colorectal cancer (%)	2.2	2.1	1.8	0.0	0.71	2.5	2.5	3.1	3.4	0.53
										
Age of final education completed ⩾20 years (%)	11.8	11.4	8.1	15.1	0.30	5.2	5.6	4.9	4.2	0.29
										
Use of laxatives (%)	4.6	21.8	46.2	60.5	<0.0001	8.5	24.1	48.5	64.3	<0.0001
										
Having frequent diarrhoea (%)	20.7	16.5	20.1	18.8	<0.0001	10.7	7.6	6.4	3.8	<0.0001

BM=bowel movement; BMI=body mass index; ANOVA=analysis of variance.

a Test for homogeneity of characteristics between categories of BM frequency, using ANOVA (age, BMI) and *χ*^2^ (other variables).

shows the baseline characteristics of the study population by BM frequency. Regardless of sex, individuals who reported infrequent BMs – compared with those who reported BMs daily or every 2–3 days – had a lower average BMI, were less likely to spend >30 min walking per day and were more likely to use laxatives.

A significant difference in the intake frequency of green leafy vegetables and in smoking status across the BM groups was observed only among women: those who reported BMs daily or every 2–3 days were more likely to consume green leafy vegetables daily and less likely to be smokers. In addition, women who reported BMs every 2–3 days were, on average, younger than those in the other BM groups. Alcohol consumption did not differ between BM groups in women.

Among men, the number that reported daily alcohol intake increased linearly with BM frequency. Male subjects who reported BMs every 2–3 days had the lowest rate of frequent diarrhoea, whereas the number of women who reported frequent diarrhoea decreased linearly with BM frequency.

During the follow-up period (average length 7.6 years, standard deviation 1.9), a total of 649 cases of colorectal cancer were identified (379 in men and 270 in women), which included 429 cases of colon cancer (225 in men and 204 in women).

Age-adjusted IRRs were calculated for colorectal and colon cancer according to BM frequency (not shown). Regardless of sex, the ratios were <1.00 for subjects who reported BMs every 2–3 days relative to those who reported daily BMs: the IRRs for colorectal cancer were 0.74 in men (95% CI=0.51–1.09) and 0.71 in women (95% CI=0.52–0.97), whereas the IRRs for colon cancer were lower in men (0.45; 95% CI=0.25–0.82) and the same in women (0.71; 95% CI=0.49–1.00). In contrast, the age-adjusted IRRs for subjects who reported highly infrequent BMs (every 6 days or less) relative to those with daily BMs were >1.00: the IRRs for colorectal cancer were 1.14 in men (95% CI=0.16–8.10) and 2.53 in women (95% CI=1.04–6.15), whereas the IRRs for colon cancer were 1.78 in men (95% CI=0.25–12.7) and 2.59 in women (95% CI=0.96–6.98).

Adjustment for potential confounding factors (as discussed above) had no significant effects on the IRRs (Table 2

**Table 2:** IRR for colorectal and colon cancer according to BM frequency by sex

		**Colorectal cancer**			**Colon cancer**		
**BM**	**Observed person-years**	**No. of cases**	**Multivariate-adjusted^a^ IRR**	**95% CI^b^**	**No. of cases**	**Multivariate-adjusted^a^ IRR**	**95% CI^b^**
*Men*							
⩾1per day	175 485	346	1.00		211	1.00	
Every 2–3 days	18 335	30	0.77	0.53–1.12	11	0.46	0.25–0.85
Every 4–5 days	1515	2	0.56	0.14–2.26	2	0.93	0.23–3.75
Every 6 days or less	321	1	1.16	0.16–8.27	1	1.86	0.26–13.4
							
*Women*							
⩾1per day	196 472	204	1.00		155	1.00	
Every 2–3 days	72 891	51	0.71	0.52–0.97	38	0.70	0.49–0.996
Every 4–5 days	8937	10	1.12	0.59–2.11	7	1.01	0.47–2.17
Every 6 days or less	1880	5	2.47	1.01–6.01	4	2.52	0.93–6.82

IRR=incidence rate ratios; BM=bowel movement; BMI=body mass index.

a Adjusted for age, BMI, intake frequency of green leafy vegetables, daily alcohol drinking, current smoking status, time spent for walking per day, family history of colorectal cancer and education.

b CI: confidence interval.

). Furthermore, even after excluding the first 3 years of follow-up, there was a lower risk of colorectal or colon cancer in women who reported BMs every 2–3 days relative to those who reported daily BMs: the multivariate-adjusted IRRs were 0.64 for colorectal cancer (95% CI=0.43–0.96) and 0.68 for colon cancer (95% CI=0.43–1.05). Increased risks of colorectal and colon cancers were also observed in association with highly infrequent BMs (every 6 days or less), although they were not statistically significant.

Table 3

**Table 3:** IRR for colorectal and colon cancer according to laxative use and susceptibility to diarrhoea

		**Colorectal cancer**			**Colon cancer**		
	**Observed person-years**	**No. of cases**	**Multivariate-adjusted^a^ IRR**	**95% CI^b^**	**No. of cases**	**Multivariate-adjusted^a^ IRR**	**95% CI^b^**
*Men*							
Laxative use							
No	155 068	292	1.00		170	1.00	
Yes	10 015	33	1.28	0.89–1.86	20	1.31	0.81–2.11
Susceptibility to diarrhoea							
Normal	143 808	285	1.00		168	1.00	
Having frequent diarrhoea	35 775	68	1.08	0.82–1.41	40	1.08	0.76–1.53
							
*Women*							
Laxative use							
No	206 189	183	1.00		137	1.00	
Yes	33 097	41	1.20	0.85–1.69	33	1.26	0.86–1.85
Susceptibility to diarrhoea							
Normal	230 880	224	1.00		173	1.00	
Having frequent diarrhoea	23 417	26	1.18	0.79–1.78	16	0.95	0.57–1.59

IRR=incidence rate ratios; BMI=body mass index.

a Adjusted for age, BMI, intake frequency of green leafy vegetables, daily alcohol drinking, current smoking status, time spent for walking per day, family history of colorectal cancer and education.

b CI: confidence interval.

shows the associations between laxative use, susceptibility to diarrhoea and colorectal or colon cancer risk. There were weak nonsignificant positive associations between laxative use and cancer risk in both men and women, but no association between cancer risk and frequent diarrhoea.

## DISCUSSION

This is the first prospective study, to our knowledge, that has reported a significant association between BM frequency and colorectal cancer risk. Infrequent BMs were associated with a significantly increased risk of colorectal cancer and a marginally increased risk of colon cancer in women. A similar, but nonsignificant, association was found in men. These results were not altered by adjusting for potential confounding factors or excluding the first 3 years of follow-up from the analysis, which indicated that the effects of the cancers themselves on bowel habits were not responsible for the associations.

These results support the findings of recent case–control studies and of the meta-analysis carried out by Sonnenberg and Müller (1993), which reported a significantly increased risk of colorectal cancer in association with constipation or infrequent BMs. However, the findings from the Nurses' Health Study in the United States – only one published prospective data on the association between BM frequency and female colorectal cancer risk (Dukas *et al*, 2000) – did not support an association between infrequent BMs and the risk of colorectal cancer. One possible reason for the discrepancy between these results and those of the present study is that different criteria were used to define ‘infrequent BM’. The Nurses' Health Study defined this as an average frequency of ‘every third day or less’. However, in the present study, a significantly increased risk of colorectal and colon cancer was found only in subjects who reported BMs every 6 days or less relative to those reporting daily BMs. Therefore, we suggest that only highly infrequent BMs elevate the risk of colorectal cancer.

Daily BMs were found to increase the risk of colorectal and colon cancer compared with BMs every 2–3 days, in both men and women. This observation is in line with the results of a previous case–control study carried out in Japan (Kato *et al*, 1993). However, the Nurses' Health Study (Dukas *et al*, 2000) found no difference in colorectal cancer incidence between subjects who reported ⩾2 BMs per day and those who reported BMs once per day (multivariate-adjusted IRR=0.89, 95% CI=0.65–1.20). Unfortunately, limitations of the questionnaire used in the present study precluded us from determining the risk associated with ⩾2 BMs per day. On the basis of the combined findings of these studies, we speculate that subgroups that have highly frequent BMs might be at an increased risk of colorectal cancer. Experimental studies have reported elevated levels of prostaglandin E_2_ (PGE_2_) in the gastrointestinal tract in many diarrhoeal states (Burakoff and Percy, 1992), and increased levels of PGE_2_ might be associated with carcinogenesis in the large intestine (Reddy *et al*, 1993). We did not observe a significant association between self-reported susceptibility to diarrhoea and colorectal cancer risk, and the results of previous epidemiological studies (case–control studies only) were inconsistent (Dales *et al*, 1979; Kune *et al*, 1987). Similar to ‘constipation’, the definition of ‘diarrhoea’ is equivocal. Some case–control studies have suggested that ‘soft’ or ‘loose’ faeces might increase the risk of colorectal cancer (Kato *et al*, 1993; Inoue *et al*, 1995). To have a conclusion, additional data on factors such as faecal consistency should be collected and analysed together with data on susceptibility to diarrhoea and BM frequency.

A weak nonsignificant positive association was found between laxative use and the risk of colorectal cancer in both men and women. Previously, the meta-analysis of Sonnenberg and Müller (1993) revealed a significant 46% increase in the risk of colorectal cancer associated with the use of laxatives. On the other hand, recent case–control studies (Jacobs and White, 1998; Nascimbeni *et al*, 2002; Roberts *et al*, 2003) and a prospective study (Dukas *et al*, 2000) found no relationship between these factors – although Dukas *et al* suggested that some types of laxative might influence intestinal pH and the metabolism of intestinal flora, thereby modifying colorectal cancer risk. The effects of laxative type were not investigated in the present study because of limitations of the questionnaire. Further prospective studies investigating the types of laxative and duration of use will be necessary to resolve this question.

The risk of rectal cancer was not analysed independently because of the small number of cases in the study group. Larger-scale prospective studies will be necessary to reveal the effects of bowel habits on the development of cancers of the large intestine at specific sites.

There were some limitations to the scope of the present study. For example, although the main risk factors for colorectal cancer were adjusted for in the analysis, other factors such as aspirin use and hormone replacement therapy in women might have confounded the results. Also, bowel habits were evaluated only through a self-reported questionnaire that was administered once at the baseline; the reproducibility and validity of the responses of subjects were therefore not confirmed.

In conclusion, this study shows that highly infrequent BMs can increase the risk of colorectal cancer in both men and women. Highly frequent BMs may also enhance this risk. Further prospective studies are needed to confirm our findings and to clarify the risk associated with BMs for colorectal cancer by subsite.
